# Establishing a Partnership to Support an HIV Prevention Intervention for Latina Women in South Florida (United States of America)

**DOI:** 10.17533/udea.iee.v42n3e10

**Published:** 2024-10-19

**Authors:** Evelyn Iriarte, Rosina Cianelli, Joseph P. De Santis, Giovanna De Oliveira, Jose G. Castro, Maria Jose Baeza, Sophia Thomas, Shanelle Hodge, Susan Rubio Rubio Rivera

**Affiliations:** 1 Ph.D, MSN, RN. Assistant Professor. Email: evelyn.iriarteparra@cuanschutz.edu. Corresponding author. https://orcid.org/0000-0002-9618-7898 University of Colorado USA evelyn.iriarteparra@cuanschutz.edu; 2 Ph.D, MPH, RN, IBCLC, FAAN. Professor. Email: rcianelli@miami.edu. https://orcid.org/0000-0002-4294-5569 University of Miami USA rcianelli@miami.edu; 3 PhD, ARNP, ACRN, FAAN. Associate Professor. U.S. Email: jdesantis@miami.edu. https://orcid.org/0000-0001-9144-7876 University of Miami USA jdesantis@miami.edu; 4 Ph.D, MSN, ARNP, ANP-C, PMHNP-BC. Clinical Associate. Email: g.deoliveira@umaimi.edu. https://orcid.org/0000-0003-4776-4342 University of Miami USA g.deoliveira@umaimi.edu; 5 MD. Professor of Clinical. Email: jcastro2@med.miami.edu. https://orcid.org/0000-0002-8642-1724 University of Miami USA jcastro2@med.miami.edu; 6 Ph.D, RN. Impact scholar post-doctoral fellow. Email: bmariajo@med.umich.edu. https://orcid.org/0000-0001-8594-9095 University of Michigan USA bmariajo@med.umich.edu; 7 MSN, MBA, MM. Ph.D candidate. Email: sot10@miami.edu. https://orcid.org/0000-0002-5119-7718 University of Miami USA sot10@miami.edu; 8 RN. Ph.D candidate. Email: sxh1162@miami.edu. https://orcid.org/0009-0007-4249-436X University of Miami USA sxh1162@miami.edu; 9 MSW. Executive Director at M.U.J.E.R. Email: susan@mujerfla.org. https://orcid.org/0009-0006-4432-4201 Women United in Justice, Education & Reform M.U.J.E.R USA susan@mujerfla.org; 10 University of Colorado College of Nursing, Aurora, Colorado, U.S. University of Colorado University of Colorado College of Nursing Aurora Colorado USA; 11 School of Nursing and Health Studies, University of Miami, Coral Gables, Florida, U.S. University of Miami School of Nursing and Health Studies University of Miami Coral Gables Florida USA; 12 Miller School of Medicine, Medical Campus, University of Miami, Coral Gables, Florida, U.S. University of Miami Miller School of Medicine Medical Campus University of Miami Coral Gables Florida USA; 13 Center for Global Health Equity, University of Michigan, Ann Arbor, Michigan, U.S. University of Michigan Center for Global Health Equity University of Michigan Ann Arbor Michigan USA; 14 Mujeres Unidas en Justicia, Educación, y Reforma (Women United in Justice, Education & Reform), Naranja, Florida, U.S. Women United in Justice, Education & Reform Mujeres Unidas en Justicia, Educación, y Reforma (Women United in Justice, Education & Reform) Naranja Florida USA

**Keywords:** community-based participatory research, HIV, Hispanic or Latino women, investigación participativa basada en la comunidad, VIH, hispánicos o latinos, mujeres., pesquisa participativa baseada na comunidade, HIV, hispânico ou latino, mulheres

## Abstract

**Objective.:**

To describe practices used in the formation of a community-based participatory research (CBPR) partnership between M.U.J.E.R., a community-based organization located in South Florida, and the School of Nursing and Health Studies at the University of Miami (United States of America). The purpose of this partnership was to adapt SEPA -*Salud, Educación, Prevención, Autocuidado*; Health, Education, Prevention, Self-care, in English- into SEPA+PrEP -Salud, Educación, Prevención, Autocuidado + Profilaxis Pre-exposición; Health, Education, Prevention, Self-care + Pre-exposure prophylaxis, in English) to facilitate CBPR focused on HIV prevention among Latina women.

**Methods.:**

Our community-based participatory research (CBPR) partnership blends multiple perspectives from community partners (community advisory board, community centers), clinical experts, cisgender heterosexual Latina members of the community, and academic/research members.

**Results.:**

Partnering practices included (i) developing a collaborative and trusting partnership, (ii) relationship building and attending to power dynamics, (iii) building capacity through mutual learning, (iv) conducting research to address barriers to HIV prevention among Latina women, and (v) implementation of knowledge gained in future CBPR.

**Conclusion.:**

The long-term success of our CBPR partnership should be measured by the capacity developed within the community and the successful implementation of community programming. Key Intentional implementation of CBPR partnership practices, tailored to academic and community institutions’ unique needs, can result in high-trust, long-term relationships.

## Resumen

**Objetivo.:**

Describir las prácticas utilizadas en la formación de una colaboración de investigación participativa basada en la comunidad (CBPR) entre MUJER, una organización comunitaria ubicada en el sur de Florida y la Facultad de Enfermería y Estudios de la Salud de la Universidad de Miami. El propósito de esta colaboración fue adaptar SEPA -Salud, Educación, Prevención, Autocuidado- a SEPA+PrEP -Salud, Educación, Prevención, Autocuidado + Profilaxis Preexposición- para facilitar la prevención del VIH entre mujeres latinas.

**Métodos.:**

Nuestra colaboración combina múltiples perspectivas de socios comunitarios (junta asesora comunitaria, centros comunitarios), expertos clínicos, miembros latinos heterosexuales cisgénero de la comunidad y miembros académicos/de investigación.

**Resultados.:**

Las prácticas de colaboración incluyeron (i) desarrollar una asociación colaborativa y de confianza, (ii) construir relaciones y atender las dinámicas de poder, (iii) desarrollar capacidades a través del aprendizaje mutuo, (iv) realizar investigaciones para abordar las barreras a la prevención del VIH entre mujeres latinas, e (v) implementar el conocimiento adquirido en futuras investigaciones.

**Conclusión.:**

El éxito a largo plazo de nuestra colaboración se evidenció por la capacidad desarrollada dentro de la comunidad y la implementación exitosa de programas comunitarios. La implementación intencional de prácticas de colaboración CBPR, adaptadas a las necesidades únicas de las instituciones académicas y comunitarias, puede resultar en relaciones de alta confianza y a largo plazo.

## Resumo

**Objetivo.:**

Descrever as práticas usadas na formação de uma colaboração de pesquisa participativa de base comunitária (CBPR) entre a MUJER, uma organização comunitária localizada no sul da Flórida, e a Faculdade de Estudos de Enfermagem e Saúde da Universidade de Miami. O objetivo desta colaboração foi adaptar SEPA - Saúde, Educação, Prevenção, Autocuidado - para SEPA+PrEP - Saúde, Educação, Prevenção, Autocuidado + Profilaxia Pré-exposição - para facilitar a prevenção do HIV entre mulheres latinas.

**Métodos.:**

Nossa colaboração combina múltiplas perspectivas de parceiros comunitários (conselho consultivo comunitário, centros comunitários), especialistas clínicos, membros cisgêneros da comunidade latina heterossexual e membros acadêmicos/de pesquisa.

**Resultados.:**

As práticas colaborativas incluíram (i) o desenvolvimento de uma parceria colaborativa e de confiança, (ii) a construção de relacionamentos e a abordagem da dinâmica de poder, (iii) a construção de capacidades através da aprendizagem mútua, (iv) a realização de pesquisas para abordar as barreiras à prevenção do HIV entre as mulheres latinas, e (v) implementar os conhecimentos adquiridos em pesquisas futuras.

**Conclusão.:**

O sucesso a longo prazo da nossa colaboração foi evidenciado pela capacidade desenvolvida na comunidade e pela implementação bem-sucedida de programas comunitários. A implementação intencional de práticas colaborativas de CBPR, adaptadas às necessidades específicas das instituições acadêmicas e comunitárias, pode resultar em relacionamentos de alta confiança e de longo prazo.

## Introduction

Despite recent advancements in HIV prevention and care, HIV disproportionately affects people who self-identify as Hispanic/Latino/a/e/x (hereafter referred to as Latino) in the U.S.^1^ According to the Centers for Disease Control and Prevention (CDC) statistics, Latina women in 2019 comprised almost 16% of the female population and represented 18% of new HIV diagnoses.^2^ This rate is four times higher than non-Latina White women (5.3 vs. 1.7 per 100 000).^3^ The disproportionate impact of HIV on Latino communities in the U.S. is caused by racism, discrimination, xenophobia, homophobia, and HIV-related stigma, as well as by economic inequality, a disjointed healthcare system, and other structural barriers.^4^ "Ending the HIV Epidemic (EHE) in the United States: A Plan for America" is an initiative coordinated by the U.S. Department of Health and Human Services (HHS) to reduce new HIV infections by 90% by 2030. One EHE initiative focuses on enhancing prevention efforts, including promoting pre-exposure prophylaxis (PrEP) use and HIV testing.^5^ CDC estimates that in 2019, only 10% of eligible women in the U.S. were prescribed PrEP,^7^ with even lower estimates for Latina women.^6^^,^^7^ These disparities underscore the urgent need for targeted HIV prevention interventions for this group.

In the context of determining how to address gaps in health disparities for Latina women, we formed a community-based participatory research (CBPR) partnership between a community-based organization (CBO) located in South Florida and the School of Nursing and Health Studies at the University of Miami (UM-SONHS), United States of América. This partnership aimed to adapt SEPA (Salud [Health], Educación [Education], Prevención [Prevention], Autocuidado [Self-care]) into SEPA+PrEP (Salud [Health], Educación [Education], Prevención [Prevention], Autocuidado [Self-care]) + Pre-Exposure Prophylaxis), a culturally grounded HIV prevention intervention that adapted SEPA, an evidence-based behavioral HIV prevention intervention. SEPA+PrEP is a culturally appropriate biobehavioral HIV prevention intervention for Latina women aged 18-49 to increase knowledge and initiation of PrEP, HIV testing, and condom use. SEPA+PrEP integrated SEPA, an evidence-based behavioral HIV prevention intervention, with PrEP. SEPA is grounded in two theoretical models: Bandura’s Social Cognitive Model of Behavioral Change and Freire’s Pedagogy,^8^^,^^9^ and has been found to be effective in preventing or reducing sexual risk behaviors and depressive symptoms in randomized trials conducted among Latina women.^10^^-^^12^ The SEPA intervention was developed and tested in 2000 in Chicago by Dr. Nilda Peragallo-Montano and her research team. Further, SEPA was implemented and tested in South Florida in 2007 and again in 2012 through a partnership between M.U.J.E.R. (Mujeres Unidas en Justicia, Educacion, y Reforma (Women United in Justice, Education & Reform); and UM-SOHNS. Despite its efficacy in reducing HIV risk behaviors, SEPA, as initially conceived, did not include content and activities on PrEP, thereby motivating the adaptation of SEPA into SEPA+PrEP. In 2020, SEPA was adapted into SEPA+PrEP as part of a Miami Center for AIDS Research (CFAR) grant.^13^ SEPA+PrEP can be defined as a supporting intervention culturally designed for cisgender heterosexual Latinas.^13^ This intervention was adopted and implemented in South Florida, one of the jurisdictions where new HIV infections have been most concentrated.^14^

Nursing professionals play a pivotal role in addressing health disparities and advancing culturally sensitive healthcare practices. This article underscores the significance of a community-based participatory research (CBPR) partnership and describes practices used in the formation of a CBPR partnership to adapt SEPA into SEPA+PrEP to enhance HIV prevention among Latina women. By delving into the collaborative efforts between community organizations, clinical experts, and academic researchers, the article highlights essential practices for building trust, fostering mutual learning, and implementing culturally grounded interventions. These insights not only empower nurses to advocate for marginalized communities but also emphasize the importance of cultural competence and long-term sustainability in community health initiatives. Ultimately, this article provides insights for nursing professionals to integrate CBPR principles into their practice, ensuring effective and equitable healthcare delivery.

## Methods

**Figure 1:**
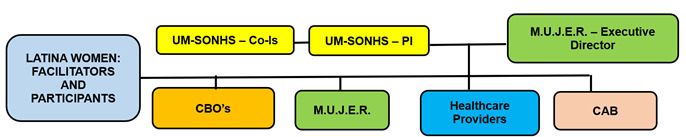
Partnership Between the M.U.J.E.R., UM-SOHNS, and Involved Stakeholders

*Note.* CAB= community advisory board; CBO= community-based organization; Co-Is= co-investigators; PI= principal investigators.

A CBPR approach was used to guide the formation and work of the partnership between the CBO and UM-SONHS to adapt SEPA into SEPA+PrEP for Latina women. CBPR is a participative approach that equitably involves community and academic stakeholders in the research process and recognizes the unique strengths that each brings.^15^^,^^16^ Established in 1994, M.U.J.E.R. is a non-profit community-based social service organization in Homestead, Florida, that aims to improve the physical and emotional wellness of the rural community.^17^ M.U.J.E.R. has earned recognition as a responsive agency by ensuring culturally sensitive services to minority communities in Homestead, South Florida.^17^ Members from UM-SONHS brought expertise in health disparities, HIV prevention and care, working with minority groups, and women’s health. Figure 1 describes the partnership and the stakeholders involved in the adaptation of SEPA into SEPA+PrEP. The parent study was approved by the Institutional Review Board of the University of Miami (IRB #20200856), and all subjects gave informed consent before data collection.

This article draws on information produced during the SEPA+PrEP partnership, including (i) reports required by the funders jointly written by community and research partners, (ii) meeting minutes, (iii) interviews with M.U.J.E.R. partners and other CBOs, (iv) focus groups with Latina women and (v) interviews with local healthcare providers. We explored how SEPA+PrEP embodied the core constructs of CBPR initially outlined by Israel *et a*l.,^15^ such as building on the strengths of the community, facilitating a collaborative exchange of knowledge and resources, and explicitly attending to power and privilege dynamics by co-creating, sharing decision-making and leveraging the diversity of experience of all partners.

## Results

### Developing a Collaborative and Trusting Partnership

SEPA+PrEP arose from partners' shared concern that Latina women in South Florida, influenced by cultural values and social determinants of health (SDoH), faced heightened HIV risk.^18^ Responding to these concerns, the research team identified a community partner with an appropriate sphere of influence in Homestead, Florida. The team intentionally sought a partner with deep, active roots in the minority women’s community, a trusted reputation, and regular active initiatives supporting community priorities. The partnership between M.U.J.E.R. and UM-SOHNS was initially established in 2006 by Dr. Nena Peragallo-Montano. Both organizations came together with a shared purpose and vision of addressing health disparities and empowering Latina women in Homestead and its surrounding communities in South Florida. This longstanding partnership has led to numerous successful projects that have positively impacted women's lives in these communities.^10^^-^^12^ By combining their resources, expertise, and dedication, M.U.J.E.R. and UM-SONHS have implemented targeted initiatives to address the specific needs of Latina women led by Dr. Peragallo-Montano's mentee, Dr. Rosina Cianelli.

**Figure 2:**
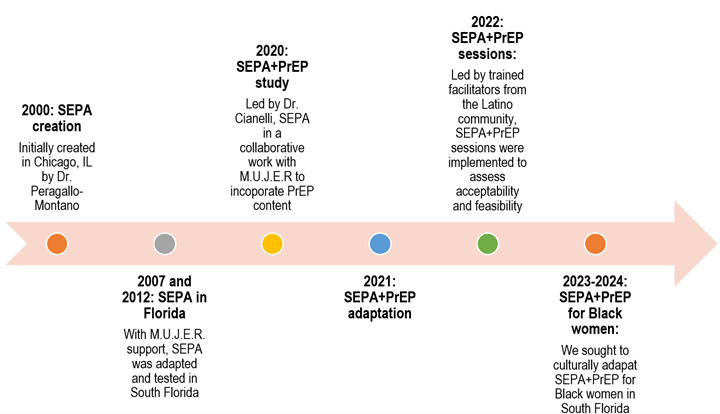
Timeline of the Partnership between M.U.J.E.R. and UM-SONHS

*Note.* M.U.J.E.R.=Mujeres Unidas en Justicia, Educacion, y Reforma (Women United in Justice, Education & Reform); SEPA= SEPA+PrEP (Salud, Educación, Prevención, Autocuidado); PrEP= Pre-Exposure Prophylaxis.

Over the years, this collaboration has taken various forms, including organizing health fairs, conducting door-to-door awareness campaigns, and obtaining grants from organizations such as the National Institutes of Health (NIH), the American Association of Colleges of Nursing (AACN), and the University of Miami CFAR. Additionally, the partnership has involved mentoring PhD students to become involved in the community (e.g., volunteering and working as research assistants) and use this site to conduct their dissertation research. The partnership has worked to identify the community's needs and priorities and has developed tailored interventions, integrating cultural elements into its approaches (community's beliefs, values, and traditions) to address the unique challenges experienced by Latina women. Throughout the collaboration, the partnership between M.U.J.E.R. and UM-SOHNS has achieved measurable outcomes and built meaningful relationships and trust within the community. By combining strengths, these entities have created a complimentary partnership that continues to evolve and adapt to the ever-changing healthcare landscape. Through collaboration, both organizations have demonstrated the power of teamwork and the possibilities that arise when common objectives, shared vision, and core values converge. Table 1 describes the partnership's vision, mission, and core values. M.U.J.E.R. and UM-SOHNS partnership has set an example for other alliances, inspiring similar relationships that strive to reduce health disparities and empower underserved communities. Figure 2 presents the timeline for the partnership between M.U.J.E.R. and UM-SOHNS.

**Table 1:** SEPA+PrEP Partnership Vision, Mission, and Core Values

Vision	Collaborate to build and provide science-based knowledge to decrease health disparities and empower Latina women to prevent HIV.
Mission	By employing rigorous research methods, our project aims to identify the specific HIV prevention requirements within the community. We tailor and test interventions that target the unique challenges Latina women encounter. In doing so, we strive to identify key stakeholders and establish partnerships that help us define and progress toward the development of a secure and trustworthy environment for Latina women. We recognize the crucial importance of providing culturally sensitive care throughout this process.
Core values	Our focus: minority women (Latina women) in South Florida Active inclusion and participation Cultural competence Empowerment Respect Confidentiality Health promotion and prevention to achieve health equity Trustworthiness Inclusion

### Relationship-Building and Attending to Power Dynamics

Team members have been mindful that resources must be equitably shared. M.U.J.E.R. and UM-SOHNS collaboratively participated in the SEPA+PrEP grant submission for Latina women, and both sites were encouraged to budget for the resources and staff needed to be successful. Though the partners are located in different county sections, team members intentionally tried to meet in person, when possible, for relationship building, leading to closer interpersonal connections and helping to build camaraderie among team members. Researchers respected that the community partner is the ultimate authority on minority women’s work and encouraged them to make autonomous decisions about their interactions with the community of Latina women and budget management. At the same time, the integrity of the research process must be maintained, and the research must comply with the Institutional Review Board (IRB) procedures in which community members were certified. Simultaneously, researchers provided the project's scientific infrastructure (e.g., scientific instruments and equipment) to ensure that outcomes were measurable. Key partnering practices for the shared contributions included working with the community partner to develop proposals and adapt recruitment and retention plans. 

Together with UM-SOHNS researchers, M.U.J.E.R. recognized the importance of involving other CBOs to expand the project's reach beyond the target population. This approach aimed to bring in different perspectives and foster a sense of community ownership. M.U.J.E.R. identified and initiated contact with two other CBOs that provide services to Latina women in Homestead, exploring their interest and collaboration capacity. M.U.J.E.R. and the research team discussed and determined which organizations would be good candidates to support the partnership. The next step involved visiting each CBO and meeting with their administrative leaders. During these meetings, researchers ensured the following aspects were discussed: (a) Project alignment with community needs: The study's objectives were clearly communicated, focusing on addressing specific community needs. This helped the researchers find organizations whose missions matched the project's goals. Understanding each organization's needs, demographics, and community relationships guided the team to identify experienced and credible partners, including those who had worked with M.U.J.E.R. before; (b) Inclusive stakeholder engagement: M.U.J.E.R. and UM-SOHNS held discussions with CBO stakeholders from Grupo Amor-Homestead and The Farmworker Association of Florida (La Asociación Campesina de Florida) to gather their perspectives on collaboration. This inclusive approach ensured that community workers' desires and preferences were considered in decision-making; (c) Assessing CBO values and capacity: The project evaluated CBOs' values, priorities, and track records to align with project goals. This included reviewing past projects, community involvement, and impact, as well as assessing organizational capacities such as infrastructure, staff expertise, and available resources; (d) Building trust through engagement: M.U.J.E.R. took the lead in building trust with identified organizations Grupo Amor-Homestead and The Farmworker Association of Florida (La Asociación Campesina de Florida). UM-SONHS researchers actively participated in CBO events and initiatives, engaging in open conversations with M.U.J.E.R. These efforts were vital in establishing trust and showing the project's commitment to ongoing collaboration for the community's benefit rather than a one-time commitment.

### Building Capacity Through Mutual Learning

Building the capacity to conduct SEPA+PrEP successfully was a relevant, shared commitment by all the team members. To improve capacity building through collaborative work and reciprocal learning, weekly team meetings were held to discuss research operations, and both partners contributed to accomplishing the study activities (e.g., recruitment, enrollment, and data collection). During the analysis and reporting results phase, both teams jointly contributed. However, the research team knew that partnership members have different skill sets, resulting in a need for different cross-training (e.g., human subject protection, research skills, and community engagement). Within the project's first year, community partners from M.U.J.E.R. collaborated on creating a 15-member CAB. This group met via Zoom® once a month for ten months to inform and guide the project about Latina women’s HIV prevention needs, focusing on PrEP and potential adaptations of SEPA into SEPA+PrEP. The CAB comprised community members, including representatives of community organizations, healthcare providers, community leaders, and Latina women from the community. CAB members were identified in collaboration and input from the CBOs. Researchers provided training about HIV infection, PrEP, and the SEPA intervention to the CAB members.

Ten cisgender heterosexual Latina women from the community also participated in online focus groups. Latina women discussed HIV awareness in the community and highlighted the limited knowledge about HIV and the importance of including Latino cultural norms (e.g., machismo, marianismo), HIV, PrEP, and the use of female condoms in the adaptation of SEPA into SEPA+PrEP. Women emphasized that since men are often reluctant to use condoms, PrEP could offer Latina women more control over their sexual health and choices. A panel of content experts, including local healthcare providers, researchers, and faculty with expertise in working with Latina women, HIV prevention, and intervention development, provided recommendations for adapting SEPA into SEPA+PrEP. Interviews with healthcare providers who worked with Latina women yielded insights into HIV prevention, PrEP, and intervention elements. They emphasized the limited knowledge about HIV prevention and PrEP among Latina women, the community resources available, and the crucial role of community leaders and healthcare providers in facilitating PrEP access. One provider noted that accurate information about PrEP, delivered by a trusted source, could greatly improve its acceptance. “[Talking about PrEP] *I think, with the right and accurate information delivered by a trusted person, someone within the community, a leader, a thought leader, someone that is trusted.”* Two bilingual Latina women from the community were selected to facilitate the SEPA+PrEP intervention for Latina women. Before initiating the SEPA+PrEP intervention, the facilitators received 8 hours of training conducted by the researchers: 4 hours of training on HIV and PrEP and 4 hours of training on implementing the three-session SEPA+PrEP intervention. Additionally, they completed the required IRB training from the Collaborative Institutional Training Initiative^19^ so they could fully participate in the study. 

Within the second year, the community facilitators delivered the culturally adapted SEPA+PrEP intervention in three weekly 2-hour sessions with small groups of 6-10 participants, 44 Latina women in total. Sessions were held in a private space at M.U.J.E.R. and included facilitated discussions, interactive feedback, role-playing, role modeling, rehearsal of communication skills, skill-building activities, and social interaction. The content and activities proposed by Latina women in the focus groups, the CAB, the CBOs, and healthcare providers were organized around these sessions. A detailed description of the SEPA+PrEP adaptation and its feasibility and acceptability are described elsewhere.^13^ One of the core values identified in the partnership was recognizing that community partners and researchers can be both experts and novices. Likewise, having experts from the community (SEPA+PrEP facilitators) was recognized as a strength for those who participated in the SEPA+PrEP sessions. [The facilitator] *is from our community, and she speaks very well… She talked about her personal life, so we opened up a little more. I have been in other talks where it is more about the PowerPoint, and it is over… But she takes her time…to speak to you. And I loved about her.* Additionally, Latina women who participated in SEPA+PrEP emphasized that interacting with other women during the sessions allowed them to gain greater knowledge by exchanging personal histories and points of view: *All this for me was very important because we learn from each other, and some tell their stories, and that is important because there are times when that is what it is about: learning from others.*

### Research to Address Barriers to HIV Prevention Among Latina Women

Researchers and community members participated in community events to continuously identify health and service needs. The partnership took advantage of existing organizational events to recruit women for the sessions and to test the acceptability and feasibility of SEPA+PrEP through group sessions (*n*=44) and focus groups (*n*=24). The partnership suggested that building community and the inclusion of other community members, such as families (including men), would help break down cultural and gender scripts that place women at risk for HIV. Latina women in the group sessions and focus groups believed that involving more community members could help change social and cultural attitudes regarding sexuality, HIV prevention, and communication with partners. 

### Implementation of Knowledge Gained in Future CBPR

The SEPA+PrEP intervention was carefully assembled over two years, devoting time to building relationships rooted in operating agreements and defined partner roles. Participants suggested outreach strategies to involve more people to promote SEPA+PrEP in community locations such as schools, restaurants, and markets and deliberately share SEPA+PrEP content in the community. One participant, for example, suggested going to work settings: *I think you can find a place where people work and where you are allowed to go inside.* Another recommendation is to go to the local flea market: *I think, at the flea market, you will have your table with the things you want to show, and people will start coming.* Over the upcoming years, new funding enabled the partnership to adapt SEPA+PrEP for Black/African American women in South Florida communities. We continue working on the second phase of assessing the effectiveness and implementation of the adapted SEPA+PrEP for Latina women. Noteworthy, this next step emphasizes the need to continue and expand this partnership.

## Discussion

SEPA+PrEP applied the principles of CBPR to address health disparities among Latina women in South Florida. Each of the organizations involved in this partnership had unique expertise but shared common interests in decreasing HIV disparities among minority women. Openness to a partnership with other CBOs led by M.U.J.E.R. (Grupo Amor-Homestead and The Farmworker Association of Florida (La Asociación Campesina de Florida) was key to achieving our common goal. The belief that community members' involvement in the development, adaptation, and management of community initiatives was vital to future implementation and sustainability. The sense of identity and emotional connection to others is essential to building community.^15^ In the CBPR approach, this identification fosters a collaborative energy to develop an effective relationship between the different entities of the partnership. In this process, a shared recognition of HIV disparities among minority women bridged the strengths of M.U.J.E.R. and the research team. The challenge of providing a community solution to address the higher risk for HIV infection among the underserved communities in Homestead became the unit of identity. This common objective was the cornerstone to facilitate an ongoing collaboration, which evolved by engaging in mutual dialogue until a trusted partnership was reached. This dialogue was made possible primarily through investing time and effort in identifying common goals and visions, honoring agreed commitments responsibly and effectively, and providing fair distribution of roles. Such dedication resulted in the successful adaptation of SEPA into SEPA+PrEP for Latina women, which increased the sense of community and identity, paving the way for the subsequent new project. 

The longstanding community-academic partnership between M.U.J.E.R. and UM-SOHNS facilitated the identification of each party’s strengths and resources and the interplay of these resources to result in a successful collaboration. As previously shown in the literature,^20^^,^^21^ researchers combined their research infrastructure with UM-SOHNS's expertise in minority women's work and their concern about a specific healthcare issue. M.U.J.E.R. also possessed local and institutional knowledge and expertise in community perspectives, key gatekeepers, common languages, and a source of trust for the community, which were critical factors in successfully reaching the targeted communities and developing the proposed projects. By respecting and building on the community's strengths and resources, the community-academic team, and putting the effort together, a critical outcome was achieved: the SEPA+PrEP intervention was feasible and acceptable for Latina women.^13^ Racial/ethnic disparities in HIV prevalence within U.S. Latino communities are influenced by SDoH, impacting HIV prevention and treatment.^22^ SEPA+PrEP aimed to address these inequalities by empowering minority communities with knowledge. Through this intervention, participants forged connections with each other and with community partners, creating a reciprocal learning environment.^15^ This collaborative effort between [M.U.J.E.R. and UM-SOHNS was guided by an ecological perspective, recognizing the multifaceted influences on individual and community health.^23^

The SEPA+PrEP intervention delved into various factors affecting the Latino community, including behavioral changes crucial for HIV and sexually transmitted infections prevention (such as partner negotiation skills, condom use, and PrEP). Additionally, environmental factors like social, economic, and physical aspects were explored before and during the study. In collaboration with community partners, the research team was well-versed in these factors specific to minority communities. The interdisciplinary CBPR team assessed how these communities' socioeconomic and physical factors interact. Our stakeholders (CAB, Latina women, and healthcare providers) worked closely with the academic team to understand and address these factors while implementing the intervention. This collaborative effort ensured that the SEPA+PrEP intervention was tailored to the specific needs and circumstances of the communities involved, fostering a holistic approach to HIV prevention and health promotion. Community partners and academic researchers were closely involved in each step of the research process during SEPA+PrEP. This included being involved from the study's inception to the dissemination of findings. Cyclical regular reviews of the research were conducted to ensure that everyone’s input was included and that the study followed the process agreed upon at the beginning of the research. 

This study's findings suggest meaningful ways to share research from the partnership between CBOs and academic institutions. Dissemination of findings is essential to CBPR^25^ as it supports the partnership's sustainability. It is crucial to focus on this during dissemination to ensure the partnership continues beyond the study.^24^^,^^25^ Additionally, sharing research can benefit both CBOs and academic institutions and promote mutual growth. For instance, academic institutions gain access to client populations for future research, while CBOs can prioritize meeting their clients' health needs, potentially leading to grants and publications.^24^^,^^25^ CBPR connects researchers with CBO clinicians, allowing for collaborative knowledge creation benefiting the community.^26^ Setting mutual goals for health impact is crucial, especially when incorporating findings into clinical practice.^24^ Likewise, CBPR can inspire future research ideas. Evaluations should be rigorous and practical, helping identify and overcome partnership gaps and barriers.^27^ The model can also guide future partnerships with other CBOs through presentations in the community, sparking new research ideas and collaborations.

Implications for Nursing. The establishment of the SEPA+PrEP intervention partnership underscores the vital role of nursing professionals in bridging gaps in HIV prevention and care among Latina women in South Florida. Through the intentional application of CBPR principles, this initiative not only adapted the SEPA intervention to include PrEP but also cultivated a collaborative environment involving community partners, clinical experts, and academic researchers. This partnership highlights the importance of cultural competence, mutual learning, and trust-building in developing effective, sustainable healthcare interventions. Nursing practitioners can draw valuable insights from this model to advocate for and implement similar culturally grounded interventions, ensuring equitable healthcare delivery and empowering underserved communities. The SEPA+PrEP partnership sets a precedent for nursing practice and research, emphasizing the necessity of community engagement and tailored healthcare solutions in addressing health disparities.

Conclusion. This article described practices used in the formation of a CBPR partnership involving community partners, clinical experts, cisgender heterosexual Latina members of the community, and academic/researchers who participated in the adaptation of SEPA into SEPA+PrEP. Intentional implementation of CBPR partnership practices tailored to the unique needs and priorities of the community can result in high-trust, long-term relationships. The partnership described in this article has existed for over a decade, and the application of CBPR principles has strengthened the relationship over time. The long-term success of this CBPR partnership should be measured by the capacity developed within the community and the successful implementation of community programming. 

Subventions. This study was supported by National of Institutes of Health grants to the University of Miami Center for AIDS Research grant [P30A1073961] to Savita Pahwa. This study was also supported by NIH/NCATS Colorado CTSA Grant Number UM1 TR004399. The content is solely the responsibility of the authors and does not necessarily represent the official views of the National Institutes of Health. The funders had no role in study design, data collection and analysis, decision to publish, or preparation of the manuscript. We acknowledge the entire Latina community of women who collaborated in this research project, especially Cristina Aldana and Irma Durand. We appreciate the support of M.U.J.E.R., Grupo Amor-Homestead, and The Farmworker Association of Florida (La Asociación Campesina de Florida).
